# Cytomegalovirus Reactivation During Therapy for Pediatric Acute Lymphoblastic Leukemia

**DOI:** 10.1002/jmv.70934

**Published:** 2026-04-20

**Authors:** Archana Melavarige Venkatagiri, Neha Reddy, Rhea Chandwani, Nishanth Shekar, Swathi Perampalli Manjunath, Emine Abdu Rahiman, Chiranjay Mukhopadhyay, Vasudeva Bhat Kalya

**Affiliations:** ^1^ Department of Pediatric Oncology Kasturba Medical College, Manipal Academy of Higher Education Manipal India; ^2^ Department of Microbiology Kasturba Medical College, Manipal Academy of Higher Education Manipal India

**Keywords:** acute lymphoblastic leukemia, chemotherapy‐related infections, CMV reactivation, cytomegalovirus, lymphopenia, pediatric oncology

## Abstract

Cytomegalovirus (CMV) reactivation is a recognized complication after hematopoietic stem cell transplantation, but its relevance during conventional chemotherapy for pediatric acute lymphoblastic leukemia (ALL) is poorly defined, especially in CMV‐prevalent regions. This retrospective cross‐sectional study was conducted at a tertiary pediatric oncology center in South India from January 2020 to August 2025. Children (≤ 18 years) with ALL who developed CMV reactivation during chemotherapy were included. CMV testing was performed based on clinical indications, and viral load was quantified using multiplex polymerase chain reaction. Demographic, clinical, laboratory, treatment, and outcome data were analyzed. Of 150 children treated for ALL, 29 (19.3%) experienced CMV reactivation. The median age was 6 years, with a male predominance (72.4%). Reactivation occurred predominantly during consolidation (51.7%) and maintenance (41.4%) phases. Fever was the most common presentation (75.9%). The median CMV viral load was 1.48 × 10^3^ IU/mL; 69% had viral loads > 1 × 10^3^ IU/mL. Lymphopenia (absolute lymphocyte count < 0.5 × 10^3^/mm^3^) was observed in 62% of patients. The median time to CMV PCR negativity was 14 days, and peak viral load showed a strong positive correlation with time to PCR clearance (*ρ* = 0.697, *p* < 0.001). Antiviral therapy was administered to 79.3% of patients. At last follow‐up, 86.2% were alive, with no CMV‐attributable mortality. CMV reactivation during chemotherapy for pediatric ALL is frequent in high‐prevalence settings and is associated with lymphopenia and prolonged immunosuppression. A targeted, risk‐adapted strategy for CMV testing and treatment may be preferable to universal surveillance.

**Abbreviations**
ALC: absolute lymphocyte count
ALL: acute lymphoblastic leukemia
ANC: absolute neutrophil count
CMV: cytomegalovirus
HSCT: hematopoietic stem cell transplantation

## 1 Introduction

Acute lymphoblastic leukemia (ALL) is the most common malignancy in childhood and accounts for a significant proportion of pediatric cancer–related morbidity worldwide [1]. Over the past few decades, survival outcomes in pediatric ALL have improved remarkably, with cure rates exceeding 80%–90% in high‐income settings, largely due to risk‐adapted contemporary chemotherapy protocols, improved diagnostics, and advances in supportive care [2, 3]. Despite these gains, infections continue to be a major cause of treatment‐related morbidity and mortality during cancer‐directed therapy, particularly in middle‐ and lower‐income countries, where delayed diagnosis, high infectious burden, and resource constraints further compound risk [4, 5].

In India, more than 90% of individuals are cytomegalovirus (CMV) seropositive, reflecting early childhood exposure [6, 7]. In immunocompetent hosts, primary CMV infection is usually asymptomatic or associated with mild, self‐limiting illness, and is rarely accompanied by end‐organ disease. However, in immunocompromised states—such as during intensive chemotherapy for hematological malignancies—latent CMV may reactivate, leading to rapid increases in viral load and potentially severe organ involvement, including pneumonitis, hepatitis, gastrointestinal disease, and bone marrow suppression.

The epidemiology, monitoring strategies, and management of CMV reactivation are well established in the setting of hematopoietic stem cell transplantation (HSCT), where routine surveillance and pre‐emptive therapy are standard practice. In contrast, CMV reactivation during conventional chemotherapy for pediatric ALL has received comparatively limited attention. CMV has been increasingly recognized as a potential cause of unexplained or prolonged fever in children undergoing ALL therapy; however, unlike bacterial and fungal infections, there are no standardized guidelines for screening, diagnosis, or management of CMV reactivation in this population. This gap is particularly relevant in CMV‐high prevalence regions, where the risk of reactivation may be substantial and the clinical consequences under‐recognized.

In this context, the present study aims to describe the occurrence, clinical characteristics, management, and outcomes of CMV reactivation in children undergoing therapy for ALL, and to compare baseline characteristics between patients with and without CMV reactivation. By delineating the burden and clinical characteristics of CMV reactivation in this setting, this study seeks to inform future strategies for surveillance and management in pediatric ALL, especially in resource‐limited, high‐seroprevalence regions.

## 2 Methodology

### 2.1 Study Design and Setting

This was a retrospective cohort study conducted at the Department of Pediatric Oncology of a tertiary care referral center in South India. The study period extended from January 2020 to August 2025.

### 2.2 Study Population

All pediatric patients (≤ 18 years) with ALL and who reactivated CMV during the therapy were eligible for inclusion. Patients with CMV blips, relapsed ALL and those who had HSCT were excluded. Patients were identified through institutional electronic medical records and laboratory databases.

### 2.3 Indications for CMV Testing

As per unit policy, CMV testing was performed in children undergoing ALL therapy who presented with one or more of the following clinical or laboratory features:

1. Prolonged or unexplained fever and/or unexplained cytopenia during any phase of therapy.
2. Unintentional weight loss accompanied by low absolute lymphocyte counts during maintenance.

CMV polymerase chain reaction (PCR) was not performed routinely and was undertaken only when clinically indicated.

### 2.4 CMV Detection and Definition of Reactivation

CMV viral load was detected using multiplex PCR assays performed on the Luminex MAGPIX system, using xPONENT software version 4.2. CMV reactivation was defined as the detection of CMV DNA at any level in peripheral blood by PCR. The lower limit of detection of the assay was 92 IU/mL. Any detectable CMV DNA level below 500 IU/mL was confirmed with repeat testing. Transient viremia with CMV DNA levels < 500 IU/mL that was not sustained on subsequent testing was defined as CMV blips. Patients with CMV reactivation were closely monitored clinically for evidence of CMV end‐organ disease, including CMV colitis, pneumonitis, hepatitis, and retinitis. All patients with CMV viral load > 1000 IU/mL underwent ophthalmologic evaluation with fundus examination and liver function testing. Evaluation for CMV colitis and CMV pneumonitis, including endoscopy or bronchoalveolar lavage as appropriate, was performed only in patients with relevant clinical symptoms suggestive of gastrointestinal or pulmonary involvement.

### 2.5 Data Collection

For patients with documented CMV reactivation, data were retrospectively extracted from medical records. Variables collected included demographic characteristics (age at diagnosis, gender), disease‐related factors (immunophenotype of ALL, risk stratification), and treatment‐related variables (phase of chemotherapy at the time of CMV reactivation). Laboratory parameters at the time of CMV detection, including hemoglobin level, total white blood cell count, and differential counts, were recorded. Information regarding CMV management strategies, duration of hospitalization, time to CMV PCR negativity, and treatment outcomes were also collected.

### 2.6 Statistical Analysis

Statistical analysis was performed using standard statistical software. Continuous variables were assessed for normality using the Shapiro–Wilk test. Normally distributed variables are presented as mean ± standard deviation, while non‐normally distributed variables are summarized as median with interquartile range (IQR). Categorical variables are presented as frequencies and percentages. Comparisons of baseline hematological parameters between risk groups (low‐risk vs. high‐risk ALL, phase of therapy) were performed using the Mann–Whitney *U* test for continuous variables, and Fisher's exact test was used for categorical variables. Correlations between CMV viral load and laboratory or clinical indices, including blood counts, duration of hospital stay, and time to CMV PCR negativity, were analyzed using Spearman's rank correlation coefficient.

## 3 Results

Between January 2020 and September 2025, 150 children were diagnosed with ALL and treated at our center. Among 150, 88 (58.6%) patients underwent CMV PCR at some point of time during therapy with median of 1 test per patient (IQR 1–11). Of the 150 patients, 29 (19.3%) developed CMV reactivation and were included in the study cohort; 5 patients with CMV blips were excluded. The median age at CMV reactivation was 6 years (IQR 7; range 1–17), with 72.4% of patients younger than 10 years and a male predominance (72.4%). B‐cell ALL was the most frequent subtype (65.5%), followed by T‐cell ALL (27.6%); mixed phenotype acute leukemia and T‐lymphoblastic lymphoma accounted for 3.4% each. Most patients were classified as high risk (69.0%), with the remainder belonging to standard‐ or intermediate‐risk groups. There were no significant differences in baseline demographic or disease characteristics between patients who experienced CMV reactivation and those who did not (Table 1).

**Table 1:** Baseline demographic and disease characteristics of patients with and without CMV reactivation.

Variable	CMV positive cohort (*n* = 29)	CMV negative cohort (*n* = 121)	*p*
Age (years), median (IQR)	5.81 (4.62)	5.53 (7.32)	0.759
Age group, *n* (%)			
< 10 years	23 (79.3)	85 (70.2)	0.329
≥ 10 years	6 (20.7)	36 (29.8)	
Gender, *n* (%)			
Male	22 (75.9)	71 (58.7)	0.087
Female	7 (24.1)	50 (41.3)	
Nutritional status, *n* (%)			
Normal	18 (62.1)	63 (52.1)	0.549
Thinness	11 (37.9)	53 (43.8)	
Overweight	0 (0.0)	5 (4.1)	
Type of ALL, *n* (%)			
B‐cell ALL/LBL	19 (65.5)	94 (77.7)	0.194
T‐cell ALL/LBL	9 (31.0)	26 (21.5)	
MPAL	1 (3.4)	1 (0.8)	
Risk group, *n* (%)			
Standard risk	1 (3.4)	13 (10.7)	0.074
Intermediate risk	8 (27.6)	44 (36.4)	
High risk	20 (69)	64 (52.9)	

A single episode of CMV reactivation was observed in 25 patients (86.2%), while 4 (13.8%) experienced two episodes. Reactivation occurred predominantly during consolidation (51.7%) and maintenance (41.4%) phases, with infrequent occurrence during induction and delayed intensification (3.4% each). Fever was the most common trigger for CMV PCR testing (75.9%), followed by weight loss (13.7%), thrombocytopenia (3.4%), and gastrointestinal symptoms (3.4%). The median CMV viral load at detection was 1.48 × 10^3^ IU/mL (IQR 4.63 × 10^3^), with a maximum of 1439 × 10^3^ IU/mL, demonstrating a markedly skewed distribution (Shapiro–Wilk *p* < 0.001); viral loads exceeded 1 × 10^3^ IU/mL in 69% of patients, and no virological progression occurred after treatment initiation (Table 2). No CMV end‐organ disease was observed. Routine liver function tests and fundus examinations were normal in all patients with CMV viral load > 1000 IU/mL. One patient underwent bronchoscopy for respiratory symptoms, which was negative for CMV; no patients required gastrointestinal endoscopy.

**Table 2:** Characteristics of CMV PCR testing and CMV reactivation in the CMV‐positive cohort.

Parameters	CMV‐positive cohort, *n* = 29
CMV PCR testing characteristics	
Patient undergoing CMV PCR, *n* (%)	88 (58.6)
Total number of CMV PCR performed, *n*	242
PCR test, median (range) among tested cohort	2 (1–11)
CMV reactivation, *n* (%)	29 (19.33)
Median CMV viral load (range)	1.48 × 10^3^ IU/mL (0.042 × 10^3^–1439 × 10^6^ IU/mL)
Episodes of CMV reactivation, *n* (%)	
1 episode	25 (86.2)
2 or > 2‐episode	4 (13.8)
Reason for performing CMV PCR, *n* (%)	
Prolonged fever	24 (82.7)
Weight loss	6 (20.6)
Cytopenia	1 (3.4)
Phase of ALL therapy during which CMV reactivation occurred, *n* (%)	
Induction	1 (3.4)
Consolidation	15 (51.7)
Interim maintenance	None
Delayed Intensification	1 (3.4)
Maintenance	12 (41.4)

All patients with CMV viral load > 1000 IU/mL and symptomatic patients with lower viral loads received antiviral therapy. Fifteen patients (48.3%) received intravenous ganciclovir followed by oral valganciclovir, while eight (27.6%) received valganciclovir alone. Six patients (20.7%) were managed with observation alone without specific antiviral therapy. Additionally, five patients with CMV blips were also managed conservatively without antiviral therapy. Intravenous immunoglobulin was administered in 12 patients (41.4%). Seventeen patients (58.6%) required inpatient care, with a median hospital stay of 7 days (range 3–16 days). Chemotherapy interruption attributable to CMV reactivation occurred in seven patients (24.1%), usually in the setting of concurrent cytopenia, fever, or neutropenia. Dose modification during maintenance therapy was required in five patients (17.2%) (Table 3).

**Table 3:** Treatment details and outcomes of patients with CMV reactivation.

Parameters	CMV‐positive cohort, *n* = 29
Treatment setting, *n* (%)	
Inpatient treatment (IP)	3 (10.3)
Outpatient treatment (OP)	10 (34.4)
Combination of (IP/OP)	16 (55.1)
Median duration of hospital stay	7 days
Treatment modalities and outcome, *n* (%)	
Ganciclovir + valganciclovir	15 (51.7)
Valganciclovir	8 (27.5)
IVIG	12 (41.3)
Observation	6 (20.6)
CMV end‐organ disease	None
CMV‐related mortality	None

Lymphopenia with absolute lymphocyte count ALC < 0.5 × 10^3^/mm^3^ was present in 62% of patients, while 27.5% had hemoglobin levels < 10 g/dL; other hematologic parameters were non‐normally distributed, with a median total leukocyte count of 1.9 × 10^3^/mm^3^. For analysis, standard‐ and intermediate‐risk patients were combined into a lower‐risk group (*n* = 9) and compared with high‐risk patients (*n* = 20). CMV viral load at detection and peak viral load did not differ significantly between risk groups (Mann–Whitney *U* test; *p* > 0.05). Baseline hematologic parameters were also comparable between groups. Peak CMV viral load showed no significant correlation with baseline hematologic indices. The median time to CMV PCR negativity was 14 days (IQR 7 days; range 7–90 days). A strong positive correlation was observed between peak CMV viral load and time to CMV PCR negativity (Spearman's *ρ* = 0.697, *p* < 0.001). At last follow‐up, 86.2% of patients were alive; four patients relapsed and died, with no deaths directly attributable to CMV infection.

## 4 Discussion

This study adds to the growing evidence that CMV reactivation during conventional chemotherapy for pediatric ALL‐in nontransplant settings is a clinically relevant complication in high CMV‐seropositive regions. Although CMV reactivation has traditionally been considered uncommon outside HSCT, emerging data—including our finding—indicate that CMV reactivation occurs during standard ALL therapy, particularly in the setting of prolonged immunosuppression and lymphopenia [8, 9, 10, 11].

The reported occurrence of CMV reactivation in pediatric ALL varies widely, reflecting differences in baseline seroprevalence, surveillance strategies, and diagnostic definitions. In high‐seroprevalence settings, CMV reactivation appears substantially more frequent than in Western cohorts. Şen and colleagues reported from Korea that CMV viremia was detected in over 70% of nontransplant pediatric ALL patients undergoing routine monitoring, with baseline CMV IgG positivity predicting reactivation [12]. In contrast, Abbas and colleagues observed CMV infection in only a small proportion of children when testing was symptom‐driven, suggesting that clinically overt CMV reactivation remains relatively infrequent in the absence of active surveillance [13]. Our findings lie between these extremes, supporting the concept that CMV reactivation represents a spectrum ranging from asymptomatic viremia to significant organ involvement.

A consistent observation across studies is the clustering of CMV reactivation during nonintensive phases of chemotherapy. Prior reports by Jain and colleagues, Rahbarimanesh and colleagues, and Phasuk and colleagues demonstrate that CMV disease occurs predominantly during maintenance or interim maintenance phases [8, 10, 14], a pattern mirrored in our cohort. This pattern suggests that cumulative and prolonged immunosuppression, rather than peak chemotherapy intensity, is the principal driver of CMV reactivation, likely due to sustained T‐cell dysfunction and impaired CMV‐specific immunity during maintenance therapy.

Lymphopenia emerges as the most consistent risk factor for CMV reactivation across all reviewed studies. Jain and colleagues reported lymphopenia in nearly 90% of children with CMV disease, with persistent lymphopenia associated with recurrence [8]. Phasuk and colleagues identified an ALC < 800 cells/mm^3^ as a strong predictor of high‐level CMV viremia [10], while similar associations were reported by Rahbarimanesh et al. and Abbas et al. [13, 14]. Collectively, these findings support the utility of ALC as a pragmatic marker for identifying children at increased risk of CMV reactivation during ALL therapy.

The clinical spectrum of CMV reactivation ranges from asymptomatic viremia to severe organ disease. While most CMV viremia episodes were asymptomatic in the Thai cohort reported by Phasuk et al. [10], like our study, other studies have documented serious manifestations including retinitis, pneumonitis, hepatitis, colitis, and hemophagocytic lymphohistiocytosis [8, 10, 14]. Notably, no end‐organ damage was seen in our cohort, likely reflecting early recognition and timely initiation of antiviral therapy in children with significant viral loads.

CMV‐related cytopenias pose a diagnostic challenge. Kanvinde and colleagues demonstrated that CMV accounted for over half of prolonged cytopenia episodes following standard‐dose chemotherapy and was associated with increased morbidity and treatment delays [9]. However, Bakhshi and colleagues caution against attributing cytopenias solely to CMV based on PCR positivity [15]. Our findings support a balanced approach, wherein CMV is considered in the differential diagnosis of prolonged cytopenias, but antiviral therapy is guided by clinical evidence/symptomatology/viral load rather than PCR detection alone.

Antiviral therapy with ganciclovir or valganciclovir has been shown to be effective in suppressing CMV replication and improving clinical outcomes [8, 9, 10]. However, concerns regarding ganciclovir‐induced myelosuppression and reports of spontaneous resolution of low‐level viremia highlight the need for judicious, risk‐adapted treatment strategies [15]. Reassuringly, most pediatric studies, including ours, demonstrate that CMV reactivation does not adversely impact leukemia‐related survival when appropriately recognized and managed [8, 10, 11]. Although CMV contributes to morbidity and treatment interruptions, relapse and mortality are largely driven by leukemia biology rather than CMV infection itself.

In summary, CMV reactivation during chemotherapy for pediatric ALL is common in CMV high‐prevalence regions and is strongly associated with prolonged immunosuppression and lymphopenia. Although overt CMV disease is relatively uncommon, its associated morbidity may be mitigated through heightened clinical vigilance, recognition of subtle manifestations of viremia, and timely diagnostic testing. A risk‐adapted strategy that identifies at‐risk patients such as those with unexplained fever, weight loss, or lymphopenia during vulnerable phases of therapy rather than universal surveillance or indiscriminate treatment, is likely to optimize clinical outcomes.

## Funding

The authors have nothing to report.

## Ethics Statement

This study was conducted in accordance with the ethical principles outlined in the Declaration of Helsinki and its later amendments. The study protocol was reviewed and approved by the Institutional Ethics Committee/Institutional Review Board of Kasturba Medical College and Kasturba Hospital Institutional Ethics Committee, Manipal, India (Approval No.: IEC1:87/2023).

## Consent

Given the retrospective/observational nature of the study, the requirement for informed consent was waived by the Ethics Committee. Patient confidentiality was strictly maintained, and all data were anonymized prior to analysis.

## Conflicts of Interest

The authors declare no conflicts of interest.

## Data Availability

The data that support this study are available from the corresponding author upon reasonable request.
